# Prognostic value of CT contrast staining after endovascular therapy in basilar artery occlusion stroke

**DOI:** 10.3389/fneur.2026.1801372

**Published:** 2026-04-28

**Authors:** Natascha Hohmann, Matthias P. Fabritius, Sijing Gu, Daniel Puhr-Westerheide, Philipp Lohse, Steffen Tiedt, Lars Kellert, Konstantinos Dimitriadis, Sergio Grosu, Thomas Liebig, Jens Ricke, Wolfgang G. Kunz, Osman Öcal, Paul Reidler

**Affiliations:** 1 Department of Radiology, University Hospital LMU, Munich, Germany; 2 Institute for Stroke and Dementia Research, University Hospital LMU, Munich, Germany; 3 Department of Neurology, University Hospital LMU, Munich, Germany; 4 Department of Neuroradiology, University Hospital LMU, Munich, Germany; 5 Department of Diagnostic and Interventional Radiology, Heidelberg University Hospital, Heidelberg, Germany

**Keywords:** basilar artery, computed tomography, contrast media, stroke, stroke management

## Abstract

**Background:**

Contrast staining signifies prolonged tissue absorption of iodinated contrast media following endovascular therapy (EVT) for large-vessel occlusion stroke, indicating blood–brain barrier disruption. With EVT becoming the standard treatment for treating basilar artery occlusion (BAO) stroke and considering the substantial variability in patient outcomes, our study aimed to determine the prognostic significance of post-interventional contrast staining in BAO stroke cases.

**Methods:**

We included BAO patients who received postinterventional non-contrast CT within 24 h after EVT. Expert radiologists confirmed the presence of contrast staining on CT, and its volume was quantified. Functional outcomes were assessed on the modified Rankin scale (mRS) at 90 days, and an unfavorable outcome was defined as an mRS score of ≥4. A multivariable LASSO-penalized logistic regression analysis was performed to determine the association between contrast staining and other clinical and imaging parameters with functional outcomes.

**Results:**

A total of 42 patients met the inclusion criteria (15 women, 35.7%). Contrast staining on postinterventional CT was present in 18 patients (42.9%) with a median [interquartile range / IQR] volume of 7.9 mL [3.7–14.6]. Patients with contrast staining had worse outcomes, with higher mRS scores at 90 days (median [IQR]: 6 [4–6] vs. 2 [1–4], *p* < 0.001). A multivariable LASSO analysis revealed a significant association of contrast staining with clinical outcome.

**Conclusion:**

Contrast staining on postinterventional CT after EVT for BAO is a strong predictor of unfavorable functional outcomes, outperforming other pre- and post-interventional imaging parameters.

## Introduction

Since 2020, a series of four randomized controlled trials have provided evidence on the efficacy of endovascular therapy (EVT) in the treatment of basilar artery occlusion (BAO) stroke (1–4). However, the first two studies raised concerns due to patient crossovers and mid-trial protocol adaptations, whereas the latter two studies, which demonstrated the benefits of EVT, performed rigorous selection using clinical and imaging parameters in a Chinese population. Considering these factors, the current recommendation for EVT in patients with BAO has a moderate strength and remains a topic of debate (5). Nonetheless, it can be assumed that an increasing number of patients are receiving EVT for BAO.

To support pre-interventional patient selection, retrospective studies on BAO suggest the use of CT collateral or perfusion imaging (6–8), which has shown a high accuracy in predicting outcomes after EVT, similar to what has been established in anterior circulation strokes (9–11). Only a few publications have addressed the use of post-interventional imaging for outcome prediction in BAO (12).

A common phenomenon after EVT in the anterior circulation is contrast staining (CS). This describes the occurrence of hyperdense areas in brain tissue on follow-up non-contrast CT (NCCT) imaging due to the prolonged accumulation of iodinated contrast media, which was originally applied during EVT and is associated with a poor functional outcome (13). These areas usually overlap with the topography of previously or currently ischemic tissue and result from the disruption of the blood–brain barrier (BBB) (14, 15). CS usually presents washout between 24 and 48 h and may contain a hemorrhagic component (16, 17).

To further elaborate on this imaging parameter, we aimed to assess the predictive value of NCCT contrast staining on follow-up imaging after EVT in patients with BAO using dichotomic and volumetric values in a multivariable analysis.

## Methods

### Study design and population

This retrospective study was approved by the institutional review board of LMU Munich, in accordance with the Declaration of Helsinki (2013), and the requirement for written informed consent was waived. Patients with acute ischemic stroke caused by basilar artery occlusion were selected from a consecutive cohort of prospectively enrolled patients in the German Stroke Registry (clinicaltrials.gov identifier: NCT03356392). All patients were treated for EVT between June 2015 and December 2019. We included all patients with basilar artery occlusion who had complete NCCT, single-phase CT angiography (CTA), and CTP imaging data, along with NCCT follow-up performed within 24 h after EVT. We excluded patients with incomplete imaging and premorbid modified Rankin Scale (mRS) score >3.

### Functional and clinical data

Baseline demographic and clinical characteristics were recorded. An unfavorable outcome was defined as an mRS score of > 3 at 90 days, in line with previous BAO studies. Consistent with current guidelines for basilar artery occlusion, an unfavorable outcome was defined as an mRS score of > 3 (i.e., 4–6). While the majority of anterior circulation stroke studies uses an mRS score of ≥ 3 to define an unfavorable outcome, an mRS score of > 3 is recommended for posterior circulation strokes due to their distinct clinical course and recovery patterns (18).

### Multiparametric CT imaging and analysis

The acquisition protocol and imaging analysis of admission imaging have been described in detail before. Upon admission, patients underwent a comprehensive examination using a standardized multiparametric CT protocol that included non-contrast CT, single-phase CTA, and whole-brain CTP with 10 cm z-axis coverage, all performed using a third-generation dual-source CT SOMATOM Definition Force scanner (DSCT-1: 2 × 192-slice SOMATOM Force, Siemens Healthineers, Erlangen, Germany) located in the emergency department. CTP data were analyzed using the manufacturer’s software (syngo Neuro Perfusion CT; Siemens Healthineers, Forchheim, Germany) to generate maps of cerebral blood flow (CBF), cerebral blood volume (CBV), mean transit time (MTT), and time to drain (TTD). In the imaging workflow, non-contrast CT (NCCT) is followed by arterial CT angiography (CTA) acquired in a caudocranial direction using bolus-tracking technology (CARE Bolus, Siemens Healthineers). Specifically, a region of interest (ROI) is manually positioned in the aortic arch, and image acquisition is automatically triggered once a predefined enhancement threshold of 100 Hounsfield units (HUs) is reached, with the scan proceeding toward the cranial vertex. Contrast enhancement was achieved by intravenous administration of 50 mL of non-ionic iodinated contrast agent (iomeprol; Imeron® 400 mg I/mL, Bracco Imaging, Konstanz, Germany) at a rate of 5 mL/s, followed by a 40 mL saline flush. For CTP acquisition, 40 mL of the same contrast agent was administered at a flow rate of 6 mL/s, followed by a 100 mL saline flush. The respective z-axis coverage was 201 mm for DSCT-1, 114 mm for DSCT-2, and 118 mm for SSCT. Automated exposure control (AEC) and iterative reconstruction (IR) techniques were applied to both NCCT and multiphase CTA (MP-CTA). Tube voltage settings ranged from 100 to 120 kV for NCCT and from 70 to 80 kV for MP-CTA and CTP. The images were reconstructed with a slice thickness of 2 mm (6, 19).

Assessment of contrast staining on follow-up NCCT after EVT was performed by two independent readers (M. P. F., P. R.) who were blinded to all clinical and follow-up imaging data. Discrepancies were resolved in a subsequent consensus reading.

**Figure 1:**
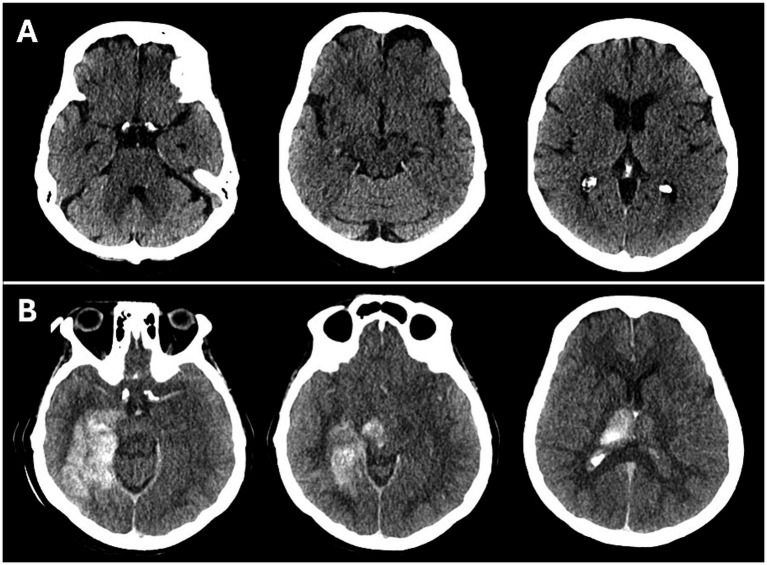
Case example of an adult patient with basilar artery occlusion: non-contrast CT before **(A)** and after **(B)** endovascular therapy. After therapy, large areas of contrast staining are seen in the PCA territory, brainstem, and thalamus. 90-day mRS was 6.

The differentiation between parenchymal CS and petechial hemorrhage on CT imaging was determined by visual assessment. Lesions were classified as contrast staining when hyperdensities exhibited imaging characteristics that were considered atypical for hemorrhage. Specifically, these were diffuse hyperdensity lesions that completely resolved or showed marked reduction within 24 h, without evidence of mass effect on follow-up, according to previous studies. (14, 20) Lesions in which hyperdensity persisted beyond 24 h and/or was accompanied by mass effect were classified as intraparenchymal hemorrhage (20). Figure 1 provides a case example. Volumes of hyperdensities due to contrast staining were measured using OsiriX v.8.0.2 (Pixmeo; Bernex, Switzerland).

### Statistical analysis

Statistical analysis was performed using R statistical and computing software, version 3.6.3.^1^ Categorical variables were reported as counts and percentages, and continuous variables as means and standard deviations or medians and ranges. Chi-square and Fisher’s exact tests or Student’s *t*-test and Mann–Whitney U-tests were performed to compare patient characteristics with or without contrast staining on follow-up imaging. Univariable analysis was used to determine factors for the occurrence of contrast staining. In a second step, significant values were used in a multivariable model to determine independent parameters. Significance was set as a two-sided *p* < 0.05.

A multivariable least absolute shrinkage and selection operator (LASSO)-penalized logistic regression model was used to identify predictors of unfavorable clinical outcomes using the R package glmnet (version 4.0). The regularization parameter was selected as the value that minimized the mean cross-validated error in 10-fold cross-validation. The LASSO allows the selection of variables by shrinking the coefficient weights for variables that are not related to the outcome to zero. Then, parameters with non-zero coefficient weights were integrated into a multivariable logistic regression analysis.

## Results

### Patient characteristics

We included 42 patients (age 74.9 ± 10.8 years, 27 men and 15 women). CS after EVT was present in 18 patients (42.9%). All observed CS lesions were parenchymal. There was no subarachnoid CS. Patients with and without contrast staining did not present significant differences in age (77.1 ± 10 vs. 73.3 ± 11.4) gender (33% vs. 38% women), occlusion location, time from symptom onset to imaging (median [IQR]: 118 min [71–209] vs. 114 min [94–138]), or admission parameters such as NIHSS or presence of coma (*p* > 0.05). The functional outcome on the mRS at 90 days was inferior in cases with contrast staining (median [IQR]: 6 [4–6] vs. 2 [0.75–4], *p* < 0.001). Patients with contrast staining presented lower rates of IV thrombolysis treatment (28% vs. 50%, *p* = 0.187) and favorable modified treatment in cerebral ischemia (mTICI) scores (76% vs. 91%, *p* = 0.373), although these findings did not reach the level of statistical significance (*p* > 0.05). Patient data are displayed in Table 1.

**Table 1:** Patient characteristics.

	Overall (*n =* 42)		No contrast staining (*n =* 24)		Contrast staining present (*n =* 18)		*P*-value
Patient data							
Age	74.9 ± 10.8		73.3 ± 11.4		77.1 ± 10		0.263
Female Sex	15	(36%)	9	(38%)	6	(33%)	0.780
Male sex	27	(64%)	15	(63%)	12	(67%)	
Time from symptom onset to imaging (min)*	116	(75–186)	114	(71–209)	118	(94–138)	0.997
Coma on admission	18	(43%)	10	(42%)	8	(44%)	0.857
Admission NIHSS*	21	(7–24)	18	(6–24)	22	(16–24)	0.252
NCCT imaging data							
pc-ASPECTS NCCT	10	(9–10)	10	(9–10)	10	(9–10)	0.251
CTA-SI							
pc-ASPECTS CTA-SI	8	(7–9)	9	(7–9)	8	(6–9)	0.169
BATMAN score	7	(5–8)	7	(6–8)	7	(6–8)	0.505
pc-CTA score	3	(2–3)	3	(2–3)	3	(2–3)	0.369
PC-CS	7	(6–8)	7	(6–7)	6	(6–8)	0.361
CTP imaging data							
CBF deficit volume [mL]	49	(21–111)	28	(17–69)	70	(48–127)	**0.013**
CBV deficit volume [mL]	5	(2–17)	4	(1–7)	18	(6–26)	**0.001**
Location of occlusion							
Vertebral arteries	14	(34.7%)	10	(42%)	4	(22%)	0.186
Proximal BA	11	(26.5%)	6	(25%)	5	(28%)	>0.999
Middle BA	19	(46.9%)	13	(54%)	6	(33%)	0.179
Distal BA	32	(73.5%)	18	(75%)	14	(78%)	>0.999
PCA	24	(57.1%)	14	(58%)	10	(56%)	0.857
PCom	1	(2%)	0	(0%)	1	(6%)	0.429
Absence of OR hypoplastic PCom	20	(47.6%)	10	(42%)	10		0.279
Treatment data							
IV thrombolysis*	17	(41%)	12	(50%)	5	(28%)	0.187
Endovascular therapy	42	(100%)	24	(100%)	18	(100%)	n.a.
Time from symptom onset to flow restoration (min)*	256	(186–334)	285	(175–346)	259	(252–260)	0.829
Favorable mTICI (≥2b)	33	(83%)	12	(50%)	5	(29%)	0.187
Volume of Hyperdensity^#^	0	(0–7.5)	0	(0–0)	7.9	(3.7–14.6)	**<0.001**
Functional data							
Premorbid mRS*	0	(0–1)	0	(0–1)	1	(0–1)	0.054
90-day mRS	4	(2–6)	2	(1–4)	6	(4–6)	**<0.001**
Cardiovascular risk factors							
Arterial hypertension*	28	(67%)	17	(71%)	11	(73%)	>0.99
Diabetes mellitus*	5	(12%)	3	(13%)	2	(13%)	>0.99
Hypercholesterolemia*	10	(24%)	6	(29%)	4	(27%)	>0.99
Atrial fibrillation*	16	(38%)	9	(38%)	7	(50%)	0.452
Smoking history*	5	(12%)	2	(11%)	3	(25%)	0.350
Etiology of stroke*							
Large artery atherosclerosis	5	(12%)	3	(13%)	2	(12%)	>0.99
Cardioembolic	21	(50%)	13	(54%)	8	(47%)	0.201
Other determined	3	(7%)	3	(13%)	0	(0%)	0.253
Undetermined	12	(29.3%)	5	(21%)	7	(41)	0.183

Values presented are no. (percentage) for categorical and median (interquartile range) or mean ± standard deviation for continuous variables based on normality distribution. Proportion analysis tests for categorical variables were performed using Fisher’s exact test or chi-square test. Non-parametric tests for ordinal and continuous variables were performed using the Mann–Whitney U-test or t-test. BA, basilar artery; BATMAN, basilar artery on computed tomography angiography; CBF/CBV, cerebellar blood flow/volume; CTA-SI, CT angiography-source images; mRS, modified Rankin Scale; n.a., not applicable; NCCT, non-contrast CT; NIHSS, National Institutes of Health Stroke Scale; PCA, posterior cerebral artery; pc-ASPECTS, posterior circulation-Acute Stroke Prognosis Early CT Score; PC-CS, posterior circulation collateral score; pc-CTA, posterior circulation computed tomography angiography; PCom, posterior communicating artery; VA, vertebral artery. Good clinical outcome, 90-day mRS 0–3; Poor clinical outcome, 90-day mRS 4–6. Bold *p*-values indicate *p* < 0.05. * Missing values: Time from symptom onset 16/42, Admission NIHSS 5/42, iv thrombolysis 1/42, premorbid mRS 3/42, arterial hypertension 3/42, diabetes mellitus 3/42, hypercholesterolemia 6/42, atrial fibrillation 4/42, smoking history 11/42, etiology of stroke 1/42.

### Parameters of contrast staining and other imaging parameters

The volume of parenchymal hyperdense areas due to CS was (median [IQR]) 7.9 mL [3.7–14.6]). The locations included PICA territory *n =* 5, AICA territory *n =* 2, SCA territory *n =* 9, PCA territory *n =* 6, brainstem *n =* 4, midbrain *n =* 6, Thalamus *n =* 5. Pre-EVT CTP deficit volumes on different maps were larger in patients with contrast staining after EVT (median [IQR] for CBV: 18 mL [6–26] vs. 4 mL [1–6], *p* = 0.001; CBV: 70 mL [48–127] vs. 28 mL [17–69], *p* = 0.013). In multivariable regression analysis, CBF deficit volume was an independent predictor for the occurrence of CS (OR [95% CI]: 1.12 [1.02–1.26]). The univariable analysis is given in the Supplementary material. Visual scores for vascular (BATMAN, pcCTA score) or tissue status (pcASPECTS) did not differ significantly (*p* > 0.05).

Interrater reliability for the assessment of contrast staining (CS) presence was evaluated between two independent readers. The overall agreement was 83.7% (36/42 cases), with a Cohen’s kappa of 0.67 (95% CI, 0.44–0.89), indicating substantial agreement. The positive and negative agreement rates were 81.1 and 85.7%, respectively. Discrepancies were resolved through subsequent consensus reading conducted in separate sessions, with both readers reviewing the cases independently to minimize the potential bias.

### Prediction of functional outcome

**Figure 2:**
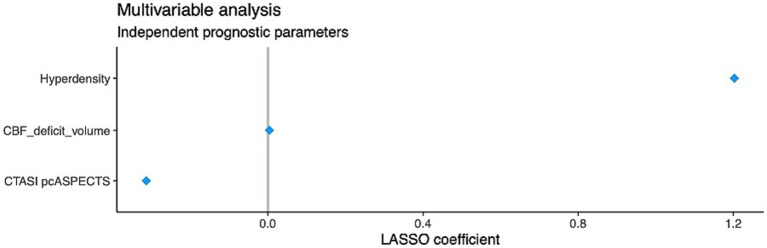
Multivariable LASSO logistic regression analysis for the prediction of unfavorable 90-day mRS.

In multivariable LASSO analysis, the occurrence of contrast staining (CS) was identified as the strongest predictor of unfavorable functional outcomes, with a LASSO coefficient of 1.20. Other relevant predictors included pcASPECTS on CTA (LASSO coefficient −0.32) and CBF deficit volume (LASSO coefficient 0.004), as illustrated in Figure 2. We observed a moderate correlation between the volume of contrast staining and 90-day mRS (Pearson’s correlation coefficient 0.44, *p* = 0.004). Among the 18 patients with CS after EVT, only 3 achieved a favorable outcome, whereas the remaining patients had unfavorable outcomes (4 patients with 90-day mRS = 4 and 11 patients with 90-day mRS = 6, *p* < 0.001).

The predictive performance of the LASSO model was strong, with an area under the receiver operating characteristic curve (AUC) of 0.88, indicating high discriminative ability. Model calibration was acceptable, with a calibration slope of 1.23, suggesting a slight tendency toward overfitting, and a Brier score of 0.14, reflecting good overall accuracy of probabilistic predictions. In a subsequent multivariable logistic regression analysis including CS, pcASPECTS, and CBF deficit volume, CS remained independently associated with unfavorable outcomes (OR 19.0, 95% CI 2.82–127.80). CBF deficit volume was also significantly associated with the outcome (OR 0.61, 95% CI 0.42–0.89), whereas pcASPECTS did not reach statistical significance (OR 0.65, 95% CI 0.21–2.01).

## Discussion

In our study, contrast staining after EVT in patients with BAO was frequent and strongly linked to an unfavorable clinical outcome. Contrast staining is positively correlated with perfusion deficit volumes but does not show an association with other baseline or treatment parameters. Therefore, we conclude that the size of ischemia is the most important factor influencing the occurrence of contrast staining after EVT. Moreover, contrast staining was the strongest predictor of an unfavorable outcome of all pre- and post-interventional parameters.

As a mechanism of contrast staining, the disruption of the BBB is assumed (13). The pathogenesis of BBB disruption is closely linked to the development of edema formation due to ischemia, which is regarded as a three-stage process: early cytotoxic, ionic edema, and vasogenic edema. The latter results from BBB breakdown due to the dysfunction of tight junctions, the neurovascular unit, and inflammatory processes. More recently, disruption of the glymphatic system has been established as another cornerstone of edema formation (21). As this system is known to remove cellular and interstitial metabolites, its dysfunction could further explain the delayed dissolvement of contrast media and its association with more severe tissue damage and unfavorable clinical outcomes (22).

While the recent RCTs raise hope for improving BAO outcomes with EVT, there is still uncertainty in the selection of patients. As RCTs do not endorse the use of perfusion imaging, which is a proven predictor for functional outcome in BAO, clear recommendations for outcome prediction are lacking (5). Although contrast staining after EVT cannot support the pre-therapeutic phase, its occurrence together with its volume is a strong and reliable indicator for an unfavorable outcome and can thereby aid in further clinical management and be used as an early outcome parameter for future studies. In the literature, Son et al. concur that the occurrence of contrast staining after EVT presents a strong predictor for unfavorable outcomes after successful recanalization in BAO (12).

This study has several important limitations that should be acknowledged. First, the overall sample size was small (*n =* 42), with only a limited number of patients exhibiting contrast staining (CS+). This small sample size reduces statistical power and increases the risk of type II error. In addition, it raises concerns regarding the stability and robustness of the estimated effects. In particular, the use of multivariable approaches such as LASSO regression and subgroup analyses in a small dataset may increase the risk of overfitting and limit the reproducibility of the findings. Therefore, the reported associations should be interpreted with caution.

Furthermore, the retrospective design may limit the generalizability of our findings and introduce the potential for selection bias and unmeasured confounding.

Second, contrast staining was assessed using single-energy CT imaging, which is commonly used in clinical practice. However, without confirmation from dual-energy CT, distinguishing contrast staining from early hemorrhagic transformation remains challenging, and misclassification cannot be excluded. This limitation may have affected the accuracy of the reported imaging findings.

Third, follow-up imaging was not performed in a standardized manner, and therefore, a systematic correlation between contrast staining and the final infarct volume was not possible. This represents an important limitation, as it restricts the ability to validate contrast staining as a reliable surrogate marker of tissue injury.

Taken together, these methodological constraints should be considered when interpreting the results of this study.

Clinical relevance for patient management remains unclear at present; however, our data suggest that contrast staining in BAO may serve as a negative predictive marker following thrombectomy, and should therefore be interpreted as hypothesis-generating rather than as a basis for actionable clinical recommendations.

Further prospective studies with larger cohorts, standardized imaging protocols, and advanced imaging techniques such as dual-energy CT are warranted to confirm and extend our observations.

## Conclusion

In conclusion, CS after EVT in BAO stroke on post-interventional CT is a strong, independent predictor of unfavorable outcome in this study, providing a potential early warning signal to identify high-risk patients and may guide therapeutic decisions, which should be investigated in future trials.

## Data Availability

The raw data supporting the conclusions of this article will be made available by the authors, without undue reservation.

## Footnotes

1
http://www.r-project.org
