# Supplementary material for: Prognostic value of CT contrast staining after endovascular therapy in basilar artery occlusion stroke
Source: Front Neurol. 2026 Apr 28;17:1801372. doi: 10.3389/fneur.2026.1801372 (PMC13160728; doi:10.3389/fneur.2026.1801372)
Supplement: Supplementary file 1 [file Data_Sheet_1.PDF]

Supplement:

Prognostic Value of CT Contrast Staining after Endovascular Therapy in Basilar Artery Occlusion Stroke

Supplemental methods:

For multivariate analysis, variable selection based on the Least Absolute Shrinkage and Selection Operator (LASSO) was performed using the R package glmnet version 4.0. The regularisation parameter was chosen as the value that minimised the mean cross-validated error in 10-fold cross-validation. The LASSO allows the selection of variables by shrinking down to zero coefficient weights for variables nonrelated to the outcome. Then, parameters with nonzero coefficient weights were integrated into a multivariable logistic regression analysis. Associated hazard ratios and 95% confidence intervals were estimated.

Univariable analysis for occurrence of contrast staining:

TABLES

Table 1. Patients characteristics							
	Overall (n=42)		Good Clinical Outcome (n=20)		Poor Clinical Outcome (n=22)		P Value
**Patient data**							
Age	74.9±10.8		71.6±11.2		78±9.7		**0.053**
Female Sex	15	(38.8%)	6	(30%)	9	(41%)	
Male sex	27	(61.2%)	14	(70%)	13	(59%)	0.461
Time from symptom onset to imaging (min)*	116	(75-186)	149	(96-240)	94	(69.5-120)	0.132
Coma on admission	18	(42.8%)	9	(45%)	9	(41%)	0.789
Admission NIHSS*	21	(7-24)	11	(4-23)	22	(16.8-24)	**0.073**
**NCCT imaging data**							
pc-ASPECTS NCCT	10	(9-10)	10	(9.75-10)	10	(9-10)	0.155
**CTA-SI**							
Location of occlusion							
Vertebral arteries	14	(34.7%)	9	(45%)	5	(22.7%)	0.191
Proximal BA	11	(26.5%)	6	(30%)	5	(22.7%)	0.729
Middle BA	19	(46.9%)	8	(40%)	11	(50.0%)	0.515
Distal BA	32	(73.5%)	13	(65%)	19	(86.3%)	0.105
PCA	24	(57.1%)	8	(40%)	16	(72.7%)	**0.032**
Absence of OR hypoplastic PCom	20	(47.6%)	6	(30%)	14	(66.6%)	**0.018**
pc-ASPECTS CTA-SI	8	(7-9)	9	(8-9.25)	7	(5.25-8)	**<0.001**
BATMAN score	7	(5-8)	7.5	(7-8)	6	(5-7)	**<0.001**
pc-CTA score	3	(2-3)	2	(2-3)	3	(2.25-3)	**0.013**
PC-CS	6.5	(6-7.75)	7	(6-8)	6	(5-7)	**0.036**
**Treatment data**							
IV thrombolysis*	17	(40.4%)	11	(55%)	6	(27.2%)	**0.085**
Endovascular therapy	42	(100.0%)	20	(100.0%)	22	(100.0%)	n.a.
Time from symptom onset to flow restoration (min)*							
Favourable mTICI (≥2b)	33	(83.3%)	18	(90%)	16	(72.7%)	0.185
**Hyperdensity**	18	(42.8%)	3	(15%)	15	(68.2%)	**<0.001**
**Volume of Hyperdensity$^{\#}$**	0	(0-7.5)	0	(0-0)	7	(0-13)	**<0.001**
**Max HU$^{\#}$**	78	(66-115)	70	(64-72)	81	(68-118)	0.185
**Mean HU$^{\#}$**	48.6±9.1		43.3±4.7		49.8±9.5		0.278

**Functional data**							
Premorbid mRS*	0	(0-1)	0	(0-1)	1	(0-1)	0.106
90-day mRS	4	(2-6)	1.5	(0-2)	6	(4.25-6)	**<0.001**
**Cardiovascular risk factors**							
Arterial hypertension*	28	(66.6%)	13	(65%)	15	(68.2%)	0.333
Diabetes mellitus*	5	(11.9%)	2	(10%)	3	(13.6%)	0.661
Hypercholesterolemia*	10	(23.8%)	6	(30%)	4	(18.2%)	0.716
Atrial fibrillation*	16	(38.1%)	8	(40%)	8	(36.4%)	>0.99
Smoking history*	5	(11.9%)	2	(10%)	3	(13.6%)	>0.99
**Etiology of stroke***							
Large artery atherosclerosis	5	(11.9%)	2	(10%)	3	(14.2%)	>0.99
Cardioembolic	21	(50%)	10	(50%)	11	(52.3%)	1.000
Other determined	3	(7.1%)	2	(10%)	1	(4.7%)	0.606
Undetermined	12	(29.3%)	6	(30%)	6	(28.6%)	0.919

Values presented are no. (percentage) for categorical and median (interquartile range) or mean±standard deviation for continuous variables based on normality distribituon. Proportion analysis tests for categorical variables were performed using the Fisher's exact test or Chi-square test. Nonparametric tests for ordinal and continuous variables were performed using the Mann-Whitney U test or t test.

Abbreviations: BA, basilar artery; BATMAN, basilar artery on computed tomography angiography; CBF/CBV, cerebellar blood flow/volume; CTA-SI, CT angiography-source images; mRS, modified Rankin Scale; n.a., not applicable; NCCT, non-contrast CT; NIHSS, National Institutes of Health Stroke Scale; PCA, posterior cerebral artery; pc-ASPECTS, posterior circulation-Acute Stroke Prognosis Early CT Score; PC-CS, posterior circulation collateral score; pc-CTA, posterior circulation computed tomography angiography; PCom, posterior communicating artery; SCA, superior cerebellar artery; VA, vertebral artery.

Good clinical outcome, 90-day mRS 0-3; Poor clinical outcome, 90-day mRS 4-6.

Bold P values indicate P<0.05.

* Missing values: Time from symptom onset 16/42, Admission NIHSS 5/42, iv Thrombolysis 1/42 Follow-up Imaging 8/49, Premorbid mRS 3/42, Arterial hypertension 3/42, Diabetes mellitus 3/42, Hypercholesterolemia 6/42, Atrial fibrillation 4/42, Smoking history 11/42, Etiology of stroke 1/42.

[#] Only for patients with hyperdensity.
